# Preferences and motivation for weight loss among knee replacement patients: implications for a patient-centered weight loss intervention

**DOI:** 10.1186/s12891-017-1687-x

**Published:** 2017-08-01

**Authors:** Christine A. Pellegrini, Gwendolyn Ledford, Sara A. Hoffman, Rowland W. Chang, Kenzie A. Cameron

**Affiliations:** 1 Department of Preventive Medicine, Feinberg School of Medicine, Northwestern University, 680 N. Lake Shore Drive, Suite 1400, Chicago, IL 60611 USA; 2 Department of Exercise Science, University of South Carolina, Discovery I, 915 Greene Street, Suite 403, Columbia, SC 29208 USA; 3 Department of Preventive Medicine, Medicine – Rheumatology, Physical Medicine & Rehabilitation, Feinberg School of Medicine, Northwestern University, 633 N. St. Clair, 18th Floor, Chicago, IL 60611 USA; 4 Department of Medicine – General Internal Medicine and Geriatrics, Department of Preventive Medicine, Feinberg School of Medicine, Northwestern University, 750 N. Lake Shore Drive, 10th Floor, Chicago, IL 60611 USA

**Keywords:** Knee replacement, Obesity, Patient preference

## Abstract

**Background:**

Most knee replacement patients are overweight/obese, yet are commonly excluded from evidence-based weight loss programs due to mobility limitations and barriers faced around the time of surgery. The purpose of this study was to identify knee replacement patient preferences for weight loss programs and qualitatively understand previous motives for weight loss attempts as well as strategies used to facilitate behavior changes.

**Methods:**

Patients who were either scheduled to have knee replacement or had one recently completed within the last 3 months were recruited to participate. Patients completed a brief weight loss program preference questionnaire assessing preferred components of a weight loss program (i.e. self-monitoring, educational topics, program duration). Qualitative interviews were completed to identify motives for and strategies used during past weight loss attempts. All interviews were transcribed, de-identified, and analyzed using constant comparative analysis.

**Results:**

Twenty patients (11 pre-operative and 9 post-operative) between 47 and 79 years completed the study (55% male, 90% White, and 85% with a BMI ≥25 kg/m^2^). Patients reported a preference for a weight loss program that starts before surgery, is at least 6 months in duration, and focuses both on diet and exercise. The majority of patients preferred to have a telephone-based program and wanted to track diet and physical activity on a smartphone application. The most common motive for weight loss mentioned by patients related to physical appearance (including how clothing fit), followed by wanting to lose weight to improve knee symptoms or to prevent or delay knee replacement. Strategies that patients identified as helpful during weight loss attempts included joining a formal weight loss program, watching portion sizes, and self-monitoring their dietary intake, physical activity, or weight.

**Conclusions:**

This study provides a preliminary examination into the motives for weight loss, strategies utilized during past weight loss attempts, and preferences for future weight loss programs as described by knee replacement patients. These results will help guide the development and adaptation of future patient-centered weight loss programs as well as help clinicians recommend targeted weight programs based on the specific preferences of the knee replacement population.

## Background

Obesity is a known contributing factor to the development and progression of knee osteoarthritis and likely leads to the need for total knee replacement [1]. The majority of knee replacement patients struggle to manage their weight, with estimates suggesting that 80–95% of patients needing a joint replacement also are overweight or obese [2, 3]. Excess weight is linked to increased risk of surgical complications [4], infections [5, 6], joint revision [6, 7], and slower recovery following knee replacement [8]. There also appears to be significant cost implications associated with excess weight, with estimates suggesting that with every unit increase in body mass index, medical costs related to knee replacement increased by $299 [9]. Due to the increased risk of medical complications, as well as the associated costs, many patients are either being denied knee replacement surgery and/or encouraged to lose weight prior to the surgery.

Despite recommendations to lose weight, fewer than 1 of 9 patients lose >5% of their body weight before surgery [10]. The mobility limitations and heightened pain patients face leading up to a knee replacement are likely major barriers to pre-operative weight loss [11]. After surgery, most patients experience significant improvements in pain and function, yet positive changes in physical activity and weight do not necessarily accompany these positive physical outcomes [12]. Small weight losses are common immediately around the time of the surgery, yet in the years after surgery, patients are less likely to have a weight loss of at least 2.5% of initial body weight [13]. Zeni and colleagues [14] estimate that 66% of patients actually *gain* weight by 2 years after surgery.

Weight loss programs for adults with knee osteoarthritis have been developed and successfully implemented [15], but few exist for knee replacement patients. Knee replacement patients are typically excluded from evidence-based behavioral weight loss programs because of the mobility limitations and additional challenges experienced immediately before or after surgery. Due to the number of barriers faced by this population [16], it may be critical to engage patients and stakeholders using a patient-centered approach to develop an effective weight loss program specifically targeted toward knee replacement patients. A patient-centered approach provides an opportunity to incorporate the patients’ perspectives and preferences within programs, which is likely to enhance patient satisfaction and adherence to treatment [17, 18]. This study used mixed methods to identify and quantify patient preferences for weight loss programs as well as qualitatively understand previous motivations and strategies for weight loss. The results of this study will help to guide the development and adaptation of future patient-centered weight loss programs as well as assist clinicians recommend targeted weight programs based on the specific preferences of the knee replacement population.

## Methods

Mixed methods were used to quantify patients preferred weight loss program components and qualitatively examine motivation and strategies for weight loss among patients before or after knee arthroplasty. All procedures were approved by the Northwestern University Institutional Review Board and patients provided informed consent prior to participation.

Patients between the ages of 40–79 years who were within 3 months of their knee replacement (either pre- or post-operatively) were recruited to participate. We chose the age range of 40–79 as it best represented the majority of the patients seeking knee replacement. Additionally, we aimed to find patients within 3 months of the knee replacement to ensure that patients would be experiencing the most intensive times relating to either the anticipation or immediate recovery of surgery. Recruitment strategies included distributing postcards within pre-operative packets and at orthopedic rehabilitation centers, reaching out to eligible patients via mail, email, and telephone, and postings on knee-related websites and forums. After obtaining informed consent, patients completed a brief demographics survey and a 17-item questionnaire assessing preferences for weight loss programs. Surveys could be completed on paper or electronically, based on patients’ preference and geographical location. The weight loss program preference questionnaire was developed by the authors to encompass components of a weight loss program that could be customized to meet the specific needs of the knee replacement population. The questionnaire included items that addressed preferences for program focus (e.g., diet only vs. diet and exercise), duration of the program, interaction with a lifestyle coach/counselor (e.g., phone, in-person, text messages), goal-setting, self-monitoring, topic areas for educational lessons (e.g. healthy eating, reducing sedentary time, post-operative expectations, etc.), and social support. The items were restricted to weight loss program options that were available for a subsequent study.

Qualitative interviews with patients were conducted by trained study team members (CP, GL) over the phone or in-person based on patient preference or geographical location. The interviews were part of a larger study examining barriers and facilitators to diet and physical activity among knee replacement patients [16]. This analysis focused on the motivations and strategies for weight loss. Specifically, patients were asked if they had ever thought about or tried to lose weight in the past. If patients had attempted weight loss, open-ended questions were asked relating to initial motivations for wanting to lose weight as well as what strategies made it easier to try to lose weight. Interviews were completed until thematic saturation was reached.

### Statistical analysis

Frequencies and averages were calculated for the demographic and weight loss preference variables. Interviews were audio recorded and later transcribed and deidentified. Two transcripts were independently reviewed by three team members (CP, KAC, GL) and initial motives and strategies for weight loss were identified. From this review, an initial codebook was developed. The remaining transcripts were reviewed individually by CP and GL, who then met regularly to discuss any new codes and to refine the codebook. All transcripts were uploaded into Dedoose (version 6.2.7) [19], and constant comparative analysis was used to organize and delineate the identified motives and strategies for weight loss [20].

## Results

Of the 24 patients screened for eligibility, 20 patients (11 pre-operative and 9 post-operative) were eligible and completed the study. The age of patients ranged from 47 to 79 years and the majority (85%) of patients were classified as overweight or obese based on self-reported height and weight. Fifty-five percent of patients were male, 90% White, and 85% had a college or graduate/professional degree. Seventeen of the 20 patients owned a smartphone, with 71% owning one for over 5 years.

### Weight loss program preferences

One post-operative patient who was not overweight/obese chose not to complete the weight loss program preferences survey; thus, survey data was available for 19 of 20 patients interviewed.

#### Program focus, duration, and timeline

Ninety-four percent of patients were interested in weight loss programs that focused on both diet and exercise. The majority of patients (*n* = 12) preferred to have a program at least 6 months in duration; Seventeen patients would want to start a weight loss program before the surgery. Only one patient indicated a preference to wait until after the surgery to initiate a weight loss program.

#### Coaching and intervention delivery preferences

The most preferred methods of interacting with a lifestyle coach were via telephone (*n* = 9), in-person (*n* = 6), or video conference (*n* = 5). Most patients (*n* = 10) were interested in having at least weekly coaching sessions. Additionally, 18 patients were open to receiving extra communication from their coach through email and/or text messaging.

#### Goal-setting

Patients expressed an interest in setting a variety of goals to promote healthy behavior change. Most commonly, patients were interested in setting goals related to fruit and vegetable intake (*n* = 16), calories (*n* = 14), and water, (*n* = 14). Furthermore, 15 knee replacement patients were interested in setting an exercise goal (e.g., brisk walk for 10 min); however only 10 patients were interested in setting a daily step goal.

#### Self-monitoring

Patients were asked to identify which diet and exercise self-monitoring methods they would use while in a weight loss program. To track their diet, using a smartphone app was the most common (*n* = 11), followed by using paper diaries (*n* = 8). Similar to diet, nearly half of the patients (*n* = 9) were also interested in using a smartphone app to track their activity. After using a smartphone app, the most commonly preferred methods to track activity were paper methods (*n* = 7) and wearing wrist-worn activity monitors (*n* = 7).

#### Social support

The most common sources of social support that patients reported as helpful while in a weight loss program were teaming up with another knee replacement patient (*n* = 7) and joining a program with a friend or family member (*n* = 5). Only 4 patients indicated that they would find it helpful to be in an online support group, meet face-to-face in groups, or use social media. Six patients were not interested in receiving any social support during a weight loss program.

#### Education

**Fig. 1:**
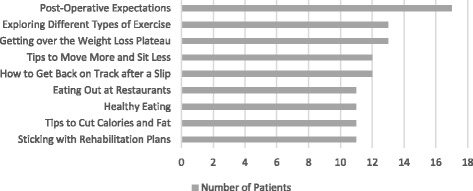
Patient Preferences for Topic Areas for Educational Lessons Delivered within a Weight Loss Program

Patients were asked to identify topics they would find helpful to learn about during a weight loss program. Highly preferred topics are presented in Fig. 1. Specifically, patients were the most interested in learning more about post-operative expectations (*n* = 17), exploring different types of exercise (*n* = 13), and “getting over the weight loss plateau” (*n* = 13).

### Motivation for weight loss

During qualitative interviews, all participants indicated previous weight loss attempts, with the most common motivation for weight loss relating to physical appearance. One pre-op female patient mentioned, “Nothing in my closet was fitting and I was getting very frustrated with it.” Another patient (pre-op, male) indicated that clothes not fitting was a cue to start losing weight: “When I’m finding it difficult to get into my clothes, my pants, then I’m thinking, ‘Well, this is not working.’” In addition to discussing clothing, the way it fits, and how it looks, several patients reported how being unhappy with their body motivated them to lose weight. “I caught my reflection in a store window in the corner of my eye…that was the breaking point…just seeing myself the way I didn’t really want to be (post-op, female).”

Many patients also mentioned being motivated to lose weight to improve their knee symptoms (e.g., pain) or to prevent or delay knee replacement: “I wanna lose weight because my knees were starting to bother me, and I figured if I lost some weight, [it would] take some strain off my knees (pre-op, male).” Another patient (post-op, female) mentioned not only the potential for pain reduction but also the hope that weight loss would prevent the imminent need of surgery: “I was just really convinced that if I lost weight my knees were going to hurt less, and I was not going to get a knee replacement at 44. I was just not [having the knee replacement] until I investigated everything, and one of the things I had in my power to actually control was my weight. So that’s what encouraged me to do it.” Once the knee replacement was scheduled, some patients were motivated to proactively lose weight to help with recovery post-surgery: “I just really want the recovery to be easier, and I know that losing weight would help (pre-op, female).”

Finally, recommendations from health care providers motivated nearly half of the patients interviewed to lose weight. Patients were encouraged to lose weight by several different providers, including orthopedic surgeons and primary care physicians: “[My primary care doctor] would say to me, ‘you’re gonna get old and not be able to move, because your weight is gonna ruin your joints.’ Guess what? He was right. If anything hearing that probably is what made me just go ahead and give [a formal weight loss program] a try (pre-op, male).” Similarly, one post-op female patient remarked, “Over time my doctor, my primary doctor has recommended that I try to lose weight because that would take some of the pressure off of my knees.” Another patient (post-op, male) shared how prior to an orthoscopic surgery, he was encouraged to lose weight: “The surgeon wanted me to lose some weight before they did the surgery so I lost 60 pounds.”

### Strategies for weight loss

Patients identified several strategies for losing weight; however the most common strategy that patients mentioned was joining a formal weight loss program, whether it was medically supervised or commercially available. One patient (pre-op, female) indicated, “That was the first time that I formally took myself to a weight loss program...It was during the time when they were doing that liquid diet. I ate one meal a day and did this liquid thing two times a day.” Similarly, another patient said, “I went to this [weight loss program] where they had this kind of milkshakes and things which were filled with vitamins and things. That got me started (post-op, female).” Other patients followed commercially available programs. “Everybody recommends [commercially available weight loss program], from doctors to therapists, everybody. I’ve done it a couple times, and it’s really effective (pre-op, male).”

In addition to formal programs, most patients mentioned how portion control and avoiding or substituting high calorie/fat food items with healthier options helped them lose weight. “I just ate half of the serving that I was served at lunch. If I put the half away in a box, when they first get the meal, I can do that, and I'm fine with that (pre-op, female).” Another patient (post-op, male) mentioned, “I started drinking tea instead [of soda], and then I tried to not snack at all, but if I did I would eat like peanut butter and celery, or carrots or something like that, cottage cheese instead of chips, cookies and cakes.”

Another strategy several patients noted included self-monitoring their dietary intake, physical activity, or weight. One pre-operative male patient indicated, “I got the little calorie book, and I write down everything I eat - breakfast, lunch, and dinner. I think that’s the only way. You just set a limited number of calories and that’s it.” Methods of self-monitoring mentioned included paper diaries, smartphone applications, and wearable activity monitors.

## Discussion

This study provides insight on both knee replacement patients’ preferences for weight loss programs and motives and successful strategies previously used during weight loss attempts. These results will help to inform future patient-centered weight management programs developed specifically for the knee replacement population. Overall, patients preferred a weight loss program at least 6 months in duration, starts prior to surgery, and focuses both on diet and exercise. The inclusion of dietary and physical activity components within a program of at least 6 months is consistent with behavioral recommendations for obesity treatment [21], with the combination of components typically resulting in greater weight losses than interventions focusing solely on diet or activity alone [22, 23]. Despite experiencing many mobility limitations and severe pain, patients reported a preference for initiation of a weight loss program prior to undergoing knee replacement. While the optimal timing to start a weight loss program in this population is unknown, initiating weight loss prior to surgery may help to reduce the risk of surgical complications and improve long-term function outcomes [8, 24].

Interestingly, although patients had an average age of approximately 65 years, most patients wanted a program that incorporated some form of technology. More patients preferred to have a telephone-based program as compared to a traditional face-to-face program; nearly all patients wanted to receive regular emails or text messages from a lifestyle coach. In addition to text messages and emails, the majority of patients would prefer to track diet and physical activity on a smartphone application. Most (*n* = 17) patients currently owned a smartphone, which may explain the strong preference for a technology-supported weight loss program. In addition, the use of smartphones for weight loss is becoming increasingly more popular [25]. Smartphone ownership observed in this sample is higher than the latest reports suggesting approximately 42% of adults over 65 own a smartphone; however, ownership continues to increase dramatically, especially in those 50 years and older [26].

More than a quarter of patients reported no interest in receiving social support during a weight loss program. This preference is surprising given that social support has been shown to be effective for weight loss in the general population [27–29]. In the knee replacement population, having a strong social support network is linked with better outcomes and function following surgery [30, 31]. Similarly, knee osteoarthritis patients who had spouses that were more empathically responsive to their pain had better physical function outcomes at 18 months as compared to those whose spouses were less empathically responsive [32]. While social support appears to play a significant role in patients’ overall physical function and recovery, it is unclear if support influences weight management in this population.

The majority of knee replacement patients, including all of the patients in the current sample, are likely to have attempted weight loss in the past [33], but the motivation behind these weight loss attempts remained unknown until now. Patients indicated that their primary motives for attempting to lose weight were related to appearance, improving knee symptoms, and recommendations to lose weight from health care professionals. These results are similar to the general population, which generally ranks appearance and health in the top reasons for wanting to lose weight [34, 35]. Additionally, while it was encouraging to hear that physicians were discussing weight loss with patients, success in weight loss and maintenance is often linked with a high sense of autonomy and intrinsic motivation [36, 37]. When weight loss is prescribed by a physician, autonomy and self-determination may be diminished and perceived as controlling, leading to a lack of enjoyment of the new eating and activity behaviors. Similarly, in the current study, over half of the patients indicated an interest in receiving education on how to get over the weight loss plateau. Patients may have had some initial success with weight loss but then struggle to maintain high levels of motivation to sustain behavior changes. Future studies may want to examine how these extrinsic motives influence success with weight loss and maintenance in knee replacement patients. Likewise, future patient-centered weight loss programs for this population may need to discuss and identify strategies to help patients identify sustainable intrinsic sources of motivation to maintain weight loss after some of the more extrinsic milestones are reached (e.g., knee pain reduced, clothing fitting).

Patients identified several strategies that helped them in previous weight loss attempts, including joining formal weight loss programs and tracking their dietary intake. Commercially available programs such as Weight Watchers and Jenny Craig have become increasingly common [38], particularly as more evidence suggests that these programs can produce significant long-term weight losses [39]. It is likely that some of the patients interviewed found these formal programs more effective than self-directed programs [40, 41]. Additionally, these programs may have provided more structure and accountability while simultaneously promoting evidence-based strategies such as self-monitoring, which was also identified as a helpful strategy by the current sample. Self-monitoring is a key component of effective behavioral weight loss interventions [42]; consistent evidence demonstrates that those who self-monitor their dietary intake have greater weight loss success than those who self-monitor less frequently [43–45].

To our knowledge, this was the first study to examine knee replacement patient preferences for a weight loss program, and the motivations and successful strategies identified by patients in the past. The study is not without limitations. First, the weight loss program preferences questionnaire was created as a tool to inform the creation of a weight loss program but was limited to favor what was feasible to provide within the bounds of the project. Though respondents were encouraged to write in free responses if no choice applied to them, some important choices may have been inadvertently omitted from the questionnaire. Despite this restriction, the data collected remain informative for the development of future weight loss programs for knee replacement patients. Second, when discussing past motivations and strategies used for weight loss during interviews, a time variable was not collected. Hence, some patients may have discussed weight loss attempts and successes occurring at a time when they were not experiencing advanced symptoms of knee osteoarthritis. Last, the sample size was small and a convenience sample of patients was recruited who were predominately white and highly educated. Therefore, the results from this study may not be generalizable to other populations.

## Conclusions

In conclusion, the results of this study provide a preliminary examination into the motives for weight loss, strategies utilized in past weight loss attempts, and preferences for future weight loss programs by knee replacement patients. Despite the need for weight management programs in this population, limited work has been done. Gaining a better understanding of patient preferences will help to inform the development of patient-centered programs, which may contribute to improved adherence to program recommendations and enhanced long-term weight loss and knee replacement functional outcomes.
